# Endoscopic gluteus maximus tendon release for external snapping hip syndrome: a functional assessment

**DOI:** 10.1186/s10195-021-00610-x

**Published:** 2021-11-10

**Authors:** Filippo Randelli, Alberto Fioruzzi, Mauro Magnani, Manuel Mazzoleni, Mohammad Elhiny, Alessio Giai Via, Olufemi R. Ayeni, Paolo Di Benedetto

**Affiliations:** 1 Hip Department (CAD), Gaetano Pini—CTO Orthopaedic Institute, University of Milan, Piazza Cardinale Andrea Ferrari 1, 20122 Milan, Italy; 2 Department of Life Health & Environmental Sciences, Unit of Orthopaedics and Traumatology, University of L’Aquila, L’Aquila, Italy; 3 Department of Orthopaedic Surgery and Traumatology, Minya University Hospital, Minya, Egypt; 4 Department of Orthopaedic Surgery and Traumatology, San Camillo-Forlanini Hospital, Rome, Italy; 5 Department of Surgery, Division of Orthopaedic Surgery, McMaster University, Hamilton, Canada; 6 Division of Orthopaedic Surgery, DAME University of Udine, ASUFC, Udine, Italy

**Keywords:** Hip endoscopy, Snapping hip, Release, Gluteus maximus muscle, Hip strength

## Abstract

**Purpose:**

The aim of this retrospective study was to investigate the clinical follow-up of patients with external snapping hip syndrome (ESHS) treated with endoscopic gluteus maximus tendon release and to compare the residual muscular strength and thigh circumference as an indirect outcome measure.

**Methods:**

Patients of all ages with external snapping hip syndrome were treated with endoscopic gluteus maximus tendon release. Outcome measures evaluated included: visual analog scale (VAS), modified Harris Hip Score (mHHS), and Non-Arthritic Hip Score (NAHS). The gluteus maximus strength and the circumference of the thigh were also evaluated.

**Results:**

Among 25 patients, 23 fulfilled the inclusion criteria and one patient was lost to follow-up. The series included 22 patients, 6 males and 16 females with a mean age of 27.9 ± 13.4 years (range 16–76 years). All patients had resolution of the snapping symptoms after the procedure. The mean follow-up was 18 ± 9.3 months. All outcomes improved in a statistically significant manner: VAS value decreased from 6.8 (range 6–8) to 0.6 (range 0–4) (*p*  < 0.001), mHHS increased from 48.6 (range 17.6–67) to 88.2 (range 67–94.6) (*p*  <  0.001), NAHS increased from 49.0 (range 21.5–66) to 90.8 (range 66–98.75) (*p*  <  0.001). A statistically significant reduction of operated limb thigh circumference compared to the contralateral side (3.7%) was also found, while there were no statistical differences regarding the strength of gluteus maximus muscles.

**Conclusions:**

Endoscopic gluteus maximus tendon release is an excellent surgical option to treat snapping hip syndrome. The evaluated muscle strength revealed no functional impairment. The significance of the limb circumference reduction has yet to be determined.

**Level of evidence:**

IV: retrospective comparative trial.

## Introduction

The snapping hip (SH), or coxa saltans, is a common clinical condition that may occur in up to 10% of the general population [1]. The prevalence appears to be slightly higher in women than in men, but sex is not a significant risk factor for external SH [2]. Although it is usually an asymptomatic condition, in some cases the snapping becomes painful, and it is called snapping hip syndrome (SHS) [3]. It is described as an audible or palpable snap [4], and three pathogenetic mechanisms are described: external snapping hip (ESH), internal snapping hip (ISH), and intra-articular snapping hip. The ESH is the most common and is caused by the snapping of the thickened iliotibial band (ITB), the gluteus maximus (GM), or a combination of these over the greater trochanter (GT) [5, 6]. Usually, the symptomatic cases are related to repetitive activities, such as ballet and running, or trauma [7].

The first treatment approach is conservative with rest, restriction of painful positions or activities, soft tissue massage, stretching exercises, non-steroidal anti-inflammatory drugs [6–8] and, in few cases, steroid injections into the area of maximal tenderness (trochanteric) [1, 9, 10]. If conservative management fails, or in case of relapse, surgical management is often advised.

In the last decade, advances in arthroscopic techniques have allowed the treatment of EHS through an endoscopic approach. Ilizaliturri et al. were the first to describe a dedicated endoscopic procedure, partially resecting the ITB from above the fasciae latae [11]. Thereafter, Voos et al. reported on an endoscopic release from below the fascia [12]. Polesello et al. have described an alternative endoscopic technique to avoid the possible iatrogenic deformity of the lateral thigh sometimes described with the previous techniques. This technique is based on the anatomical and functional correlation between the GM muscle and the ITB. The GM femoral insertion is endoscopically released close to the linea aspera, decreasing the tension of the ITB over the greater trochanter [5].

The aim of this retrospective study was to investigate the clinical follow-up of patients with ESHS treated with endoscopic gluteus maximus tendon release and to compare the residual muscular strength and thigh circumference as an indirect outcome measure.

## Materials and methods

External snapping hip syndrome was diagnosed with clinical examination: the snap was identified by palpation of the trochanteric region with flexion–extension of the hip, and by asking the patient to reproduce it voluntarily. Specific examination included the Ober test and the palpation of the trochanteric region. Standard anteroposterior, false-profile, and Dunn view radiographic projections in conjunction with magnetic resonance imaging were evaluated preoperatively to exclude concomitant intra/extra-articular pathology. All operations were performed by two senior surgeons in two University Hospitals. The retrospective investigation was approved by the Scientific Committee.

The inclusion criteria for the study were: patients of all ages; external snapping hip syndrome according to clinical and radiological findings; less than grade 2 degenerative changes according to the Tönnis scale; failure of a minimum 6 months of conservative treatment, consisting of physical therapy and steroid injections; minimum postoperative follow-up of 9 months. Exclusion criteria were chronic systemic inflammatory disease; connective-tissue disorders; femoroacetabular impingement syndrome; labral tears; hip abductor tears; dysplasia (center–edge angle of Wiberg  < 20°), axial deviation of the femoral neck (anteversion angle  > 24°, retroversion angle  > 18°, coxa valga with cervico-diaphyseal angle  > 135°, coxa vara with cervico-diaphyseal angle  < 120°); coxa profunda; or protrusio acetabuli.

At the last follow-up, the resolution of the snapping was recorded and pain was evaluated using the visual analog scale (VAS). Furthermore, the comparison of the modified Harris Hip Score (mHHS) [13] and the Non-Arthritic Hip Score (NAHS) [14] were secondary outcomes.

The extension strength (kg) of the lower limbs was measured with a dynamometer with the foot in neutral and maximus external rotation in a standing position. Every muscle contraction was tested for 5 s with a rest period of 30 s between each measure, and the whole test was performed 3 times each in order to obtain a mean value. The circumference of the thigh (cm) was measured at the midpoint between the apex of the patella and the inguinal crease.

In the period between March 2015 and November 2017, 25 patients with ESHS were treated with endoscopic gluteus maximus tendon release. A total of 23 patients fulfilled the inclusion criteria and one patient was lost to follow-up. The study group thus consisted of 22 patients, 16 women and 6 men, with a mean follow-up of 18 ± 9.3 months. The mean age of this group was 27.9 ± 13.4 years, with a range of 16–76 years.

### Statistical analysis

All statistical analysis was performed using IBM SPSS 25.0 (SPSS Inc., Chicago, IL, USA). Continuous variables are presented as mean and standard deviation with a confidence interval of 95%. A paired sample* t*-test was used to assess the difference in the patient reported outcome measures (PROMs) as well as the strength and the circumference of the operated limb before and after surgery. The percentage of outcome scores that reached the minimal clinically important difference (MCID) and Patient Acceptable Symptomatic State (PASS) between preoperative and follow-up scores were calculated [15]. A *p *value < 0.05 was considered to be statistically significant.

### Surgical technique

**Fig. 1.**
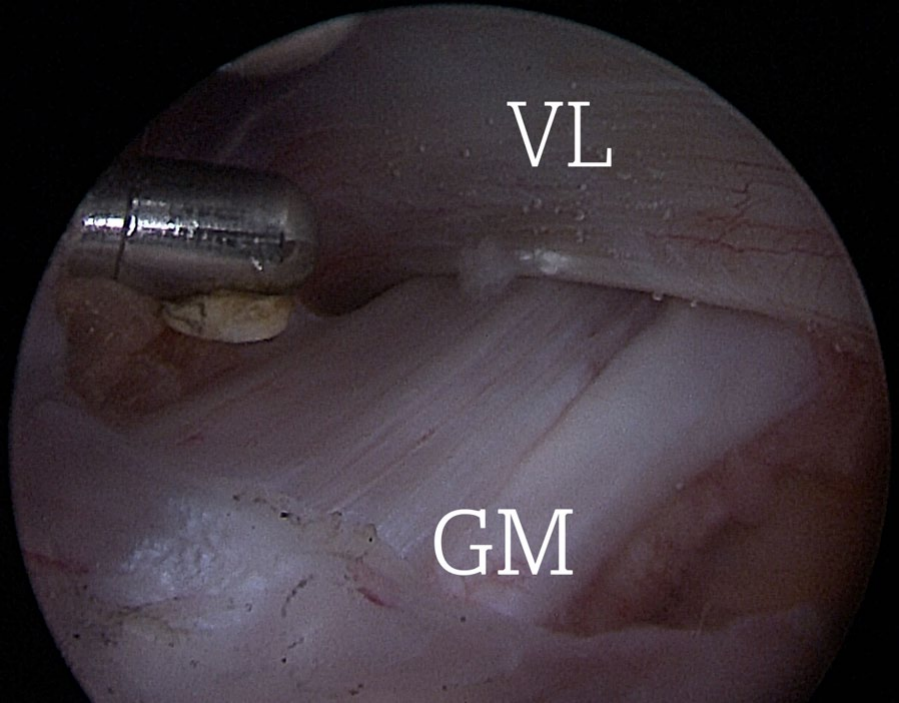
Endoscopic view of an intact femoral insertion of the gluteus maximus tendon (GM) on the linea aspera of a right femur. The tendon is located a few centimeters distal to the greater trochanter and posterior to the vastus lateralis muscle (VL)

The procedure was performed under general anesthesia with the patient placed supine on a dedicated traction operating table. The GT was marked and two portals were created: the superior trochanteric (ST) portal was located 2 cm anterior and 4 cm superior to the tip of the GT, and the second portal, the distal anterolateral accessory portal (DALA), along the axis of the femur, 10 cm below the tip of the GT. A needle was inserted through the superior trochanteric portal under fluoroscopy followed by a guidewire and the arthroscopic cannula for a standard 30° arthroscope with 4 mm diameter. Normal saline solution was pumped at low pressure (40 mmHg) to expand the virtual space between the vastus lateralis and the ITB. The DALA portal was used for direct visualization. Then, a shaver blade and a radiofrequency device were alternately introduced to create the working space. After visualizing the vastus lateralis at its superior posterior border, the femoral insertion of the GMT on the linea aspera was identified (Fig. 1).

**Fig. 2.**
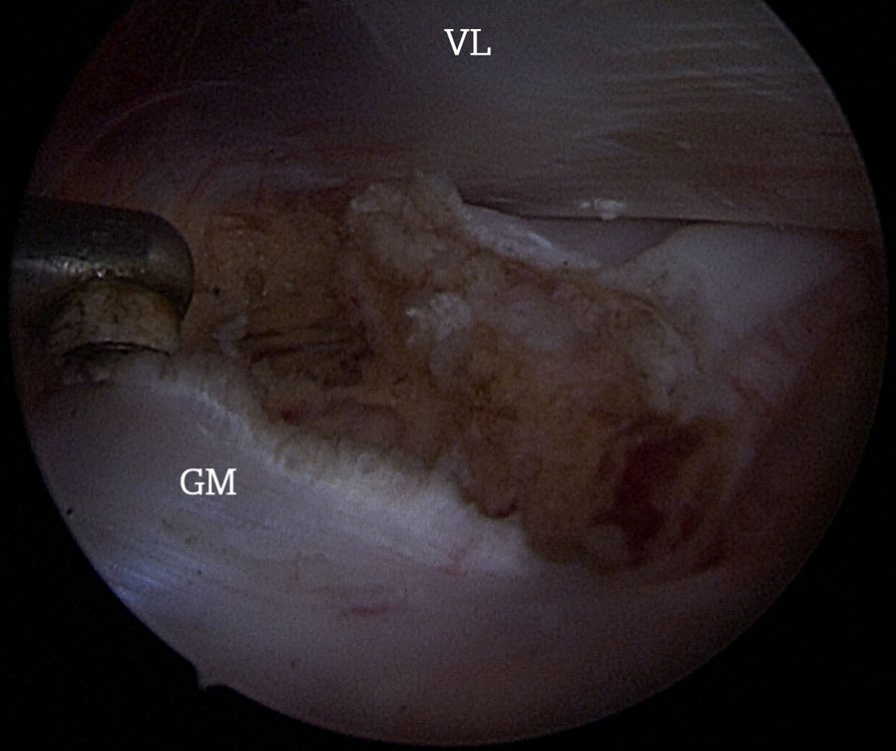
Endoscopic view after the release of the femoral insertion of the gluteus maximus tendon on the right femur. *GM* gluteus maximus; *VL* vastus lateralis

The tenotomy was performed with the radiofrequency device until a visible tendon gap was made (Fig. 2).

Once the procedure was completed, the fluid was aspirated, and the skin closed. A semi-compressive dressing was applied.

Postoperatively the patient was allowed progressively to full weight bearing with the help of two crutches for 3 weeks. Enoxaparin, 4000 UI daily, was indicated for 2 weeks for thromboprophylaxis, and Rofecoxib, 90 mg daily, for 3 weeks for heterotopic ossification prevention. The patients were encouraged to start strengthening and stretching exercises of the gluteus complex after 3 weeks from surgery.

## Results

Mean preoperative VAS was 6.8 ± 0.9 points (range 6–8). Preoperative mHHS was 48.7 ± 14.4 points with a range of 17.6–67.0 points. NAHS was 49.0 ± 9.7 points with a range of 21.5–66.0 points.

Pain and disability improved in all patients. Postoperative VAS was significantly reduced with a mean postoperative value of 0.6 ± 1.0 points (range 0.0–4.0) (*p* value < 0.001). Postoperative mHHS increased to 88.2 ± 8.6 points (range 67–94.6) (*p* value < 0.001). Postoperative NAHS increased to 90.8 ± 7.9 points (range 66.0–98.8) (*p* value < 0.001) (Table 1). MCID was reached in 100% of the patients for the mHHS and the NAHS, whereas PASS was achieved in 90.9% (20/22) of the patients for the mHHS.

**Table 1:** Comparative PROMs results

	Preop	Postop	Paired difference	*p* value
VAS	6.77	0.63	6.13	0.00
mHHS	48.66	88.25	− 39.59	0.00
NAHS	49.04	90.81	− 41.77	0.00

Extension strength difference between the healthy and the operated hip in neutral rotation showed a mean of − 3.6 ± 13.2 kg (*p* value = 0.24) and − 0.1 ± 1.2 kg in external rotation with no statistically significant difference (*p* value = 0.61). Conversely, the circumference comparison between the two thighs showed a statistically significant result (*p* value = 0.001) with a mean difference of − 1.8 ± 1.0 cm in favor of the non-operated limb (Table 2).

**Table 2:** Comparative gluteus maximus strength in extension and circumference

	Affected side	Unaffected side	Mean difference	*p* value
Strength in neutral rotation (kg)	31.24	34.86	− 3.62	0.23
Strength in external rotation (kg)	31.97	32.10	− 0.13	0.61
Thigh circumference (cm)	47.00	48.81	− 1.81	0.001

## Discussion

External snapping hip syndrome treatment has improved, in particular in the past decade, as endoscopic techniques have gained considerable popularity.

The results reported confirmed pain improvement and resolution of the snapping after endoscopic gluteus maximus tendon release, with an associated improvement in all PROMs. Concomitantly, the lower limb extension strength was maintained with no statistically significant difference compared with the contralateral leg, whereas a difference in the circumference measurement of the operated and non-operated thigh was found.

In the past, different authors have described a variety of open releases of the ITB as a solution for ESHS. Zoltan et al. [16] described 7 patients treated with an ellipsoid incision of the segment of the ITB over the GT. All patients resolved the snapping and returned to previous sport activities, although only 3 of them without any pain; one patient underwent a second surgery to obtain complete pain relief. Brignall et al. [17] treated 8 hips in 6 patients through a Z-plasty and 1 patient required revision surgery. Faraj et al. [18] described a resolution of snapping and pain with a Z-plasty and a repair of the fascia. However, 3 patients required treatments in order to relieve scar pain. Provencher et al. [19] treated 8 patients with the same open surgery as the abovementioned authors, and all the patients showed good outcomes with resolved SH and pain; however, 1 patient complained of persistent pain although there was no residual snapping.

White et al. [3] proposed a simple technique, the step-cut procedure of ITB, and treated 16 patients with a satisfying result except for pain persistence in 2 of them.

Yoon et al. [20] treated 44 patients with multiple fibrous band releases using a longitudinal incision of 8–10 cm of ITB. Subsequently, if the snap was not resolved, a GM release was added. In this series, 5 patients reported a recurrence of snapping within 3 to12 months after surgery, and 10 patients complained of residual limping or weakness. The Pedersen-Noor operation is another open technique [21] in which the authors completed a distal lengthening of the ITB of about 1.5–2 cm by Z-plasty under local anesthesia. All patients had resolved pain and SH after the procedure.

The endoscopic approach was described for the first time by Ilizaliturri et al. [11], who performed a diamond-shaped resection of the ITB in 11 patients starting from above the fascia in the subcutaneous tissue. Ten patients had good results and 1 patient was not fully satisfied. Voos et al. [12] described an ITB endoscopic resection from the peritrochanteric space starting below the fascia. Unfortunately, no clinical data were published about this technique.

In 2010 Falvey et al. [22] described, as an anatomical complex, the different structures of the lateral part of the thigh. According to their theory, fascia lata, ITB, GM, and the tensor work synergistically and ITB is tensioned anteriorly by the tensor fasciae latae (TFL) and posteriorly by the GM thanks to its insertion on the linea aspera.

Starting from this concept, Polesello et al. concluded that the GM tendon is strictly related to ITB, and its release could decrease posterior tension of the ITB. In light of this, Polesello et al. proposed a novel endoscopic technique performed with the release of the GM tendon in order to reduce the tension of the ITB. Seven out of 8 patients who underwent this surgery had their SH resolved. One patient needed a revision procedure to obtain complete resolution of symptoms. Another patient, despite resolving the ESH, presented a mild ischium snapping pain.

With the use of an almost identical surgical strategy, this study obtained excellent outcomes, with no failure or patients needing revision surgery. The strength of this study is the larger sample size of 22 patients compared with 8 in the original article; yet it is one of the largest series in the literature.

Furthermore, we obtained objective strength measurements, adding more information to the questionnaire-based outcomes. The strength of the GM muscle was assessed by a dynamometer [23] in a standardized fashion to obtain precise measurements.

Considering the lack of a validated functional outcome score for ESHS, we decided to test the patients with two different PROMs, in order to limit the potential for ceiling effects during outcome assessment.

The principal limitation of this study is the relatively small number of patients, though it expands upon the original series. Future studies should be encouraged in order to obtain more detailed results with larger sample sizes and advanced imaging such as MRI. A second concern may be the retrospective nature of the study design which could introduce unknown biases. Finally, magnetic resonance imaging changes of the muscle–tendon unit, such as fatty degeneration of the GM, were not investigated.

## Conclusions

Endoscopic gluteus maximus tendon release is an excellent surgical option to treat snapping hip syndrome. Muscle strength evaluated after the tendon release of the gluteus maximus revealed no functional impairment. The significance of the limb circumference reduction has yet to be determined.

## Data Availability

The datasets used and/or analyzed during the current study are available from the corresponding author on reasonable request.

## References

[1] Musick SR, Varacallo M (2019). Snapping hip syndrome.

[2] Lewis CL (2010). Extra-articular snapping hip: a literature review. Sports Health.

[3] White RA, Hughes MS, Burd T (2004). A new operative approach in the correction of external coxa saltans: the snapping hip. Am J Sports Med.

[4] Allen C (1995). Coxa saltans: the snapping hip revisited. J Am Acad Orthop Surg.

[5] Polesello GC, Queiroz MC, Domb BG (2013). Surgical technique: Endoscopic gluteus maximus tendon release for external snapping hip syndrome. Clin Orthop Relat Res.

[6] Yen Y-M, Lewis CL, Kim Y-J (2015). Understanding and treating the snapping hip. Sports Med Arthrosc.

[7] Potalivo G, Bugiantella W (2017). Snapping hip syndrome: systematic review of surgical treatment. HIP Int.

[8] Flato R, Passanante GJ, Skalski MR (2017). The iliotibial tract: imaging, anatomy, injuries, and other pathology. Skeletal Radiol.

[9] Nolton E, Ambegaonkar J (2018). Recognizing and managing snapping hip syndrome in dancers. Med Probl Perform Art.

[10] Idjadi J, Meislin R (2004). Symptomatic snapping hip: targeted treatment for maximum pain relief. Phys Sportsmed.

[11] Ilizaliturri VM, Martinez-Escalante FA, Chaidez PA, Camacho-Galindo J (2006). Endoscopic iliotibial band release for external snapping hip syndrome. Arthrosc J Arthrosc Relat Surg.

[12] Voos JE, Rudzki JR, Shindle MK (2007). Arthroscopic anatomy and surgical techniques for peritrochanteric space disorders in the hip. Arthrosc J Arthrosc Relat Surg.

[13] Dettoni F, Pellegrino P, La Russa MR (2015). Validation and cross cultural adaptation of the Italian version of the Harris Hip Score. Hip Int.

[14] Panico M, Gaeloto G, Bartoli V (2020). Non-arthritic Hip Score: translation, cultural adaptation and validation of the Italian version. Minerva Ortop e Traumatol.

[15] Harris JD, Brand JC, Cote MP (2017). Research pearls: the significance of statistics and perils of pooling. Part 1: Clinical versus statistical significance. Arthrosc J Arthrosc Relat Surg.

[16] Zoltan DJ, Clancy WG, Keene JS (1986). A new operative approach to snapping hip and refractory trochanteric bursitis in athletes. Am J Sports Med.

[17] Brignall CG, Stainsby GD (1991). The snapping hip. Treatment by Z-plasty. J Bone Joint Surg Br.

[18] Faraj AA, Moulton A, Sirivastava VM (2001). Snapping iliotibial band. Report of ten cases and review of the literature. Acta Orthop Belg.

[19] Provencher MT, Hofmeister EP, Muldoon MP (2004). The surgical treatment of external coxa saltans (the snapping hip) by Z-plasty of the iliotibial band. Am J Sports Med.

[20] Yoon TR, Park KS, Diwanji SR (2009). Clinical results of multiple fibrous band release for the external snapping hip. J Orthop Sci.

[21] Sayed-Noor AS, Pedersen E, Sjödèn GO (2012). A new surgical method for treating patients with refractory external snapping hip: Pedersen-Noor operation. J Surg Orthop Adv.

[22] Falvey EC, Clark RA, Franklyn-Miller A (2010). Iliotibial band syndrome: an examination of the evidence behind a number of treatment options. Scand J Med Sci Sport.

[23] Thorborg K, Petersen J, Magnusson SP, Hölmich P (2010). Clinical assessment of hip strength using a hand-held dynamometer is reliable. Scand J Med Sci Sport.

